# Cellular Function of Annexin A1 Protein Mimetic Peptide Ac2-26 in Human Skin Keratinocytes HaCaT and Fibroblast Detroit 551 Cells

**DOI:** 10.3390/nu12113261

**Published:** 2020-10-24

**Authors:** Seong Min Kim, Sang Eun Ha, Preethi Vetrivel, Hun Hwan Kim, Pritam Bhagwan Bhosale, Jung Eun Park, Jeong Doo Heo, Young Sil Kim, Gon Sup Kim

**Affiliations:** 1 Research Institute of Life Science and College of Veterinary Medicine, Gyeongsang National University, Jinju 52828, Korea; ksm4234@naver.com (S.M.K.); sangdis2@naver.com (S.E.H.); preethivetrivel05@gmail.com (P.V.); shark159753@naver.com (H.H.K.); shelake.pritam@gmail.com (P.B.B.); 2 T-Stem Co., Ltd., Gyeongsangnam-do, Changwon 51573, Korea; rari0713@t-stem.com; 3 Biological Resources Research Group, Bioenvironmental Science & Toxicology Division, Gyeongnam Branch Institute, Korea Institute of Toxicology (KIT), 17 Jeigok-gil, Jinju 52834, Korea; jdher@kitox.re.kr

**Keywords:** annexin A1, Ac2-26, anti-inflammation, anti-wrinkle, skin disease

## Abstract

Inflammation of the skin is the most common dermatological problem in human. The anti-inflammatory mediated responses of the skin cells provide a mechanism for combating these conditions. Annexin A1 (AnxA1) is one of the proteins that has been shown to have a potent anti-inflammatory effect. However, the effects and mechanisms of AnxA1 in skin keratinocyte and fibroblast have not been reported yet. In the current study, we hypothesized that Ac2-26, AnxA1 mimetic peptide, ameliorates inflammation and wrinkle formation in human skin cells. Therefore, we aimed to identify whether Ac2-26 has anti-inflammatory and anti-wrinkle effects in human keratinocyte (HaCaT) and fibroblast (Detroit 551) cells, respectively. Human HaCaT cells were stimulated by TNF-α/IFN-γ with or without Ac2-26, to identify the anti-inflammatory effect. Human Detroit 551 cells were treated with Ac2-26 to verify the anti-wrinkle effect. Initially, cell cytotoxicity was carried out in each cell line treated using Ac2-26 by MTT assay. Human MDA, IL-8, and procollagen secretion were detected by ELISA assay. The inflammatory chemokines were measured by qRT-PCR analysis. To demonstrate the mechanism, MAPK, NF-κB, JAK/STAT, and MMPs were analyzed by Western blotting. As a result, we identified that Ac2-26 significantly decreased the expression of TNF-α/IFN-γ-stimulated pro-inflammatory chemokines, including IL-1β, IL-6, IL-8, MDC, TARC, and TNF-α, by inhibiting the activation of MAPK, NF-κB, and JAK/STAT pathway in TNF-α/IFN-γ-stimulated HaCaT human keratinocytes. In addition, we also identified that Ac2-26 significantly induced collagen synthesis by generating pro-collagen, and suppressed collagen degradation by inhibiting the collagenase MMP-1 and MMP-8 expression. Collectively, these results suggest that Ac2-26 shows anti-inflammatory and anti-wrinkling effect. These effects may lead to the development of preventive and therapeutic application for inflammation-related skin disease and wrinkle formation.

## 1. Introduction

In human, the skin is the largest organ and protects from the outside environment by an epithelial barrier with abundant immune cells, such as lymphocytes and macrophages [1]. These immune cells are strongly associated with inflammatory skin responses to pathogens, but are also involved in chronic inflammatory skin diseases such as atopic dermatitis and psoriasis [2]. In addition, most aging-related skin diseases share inflammatory characteristics such as the secretion of tumor necrosis factor (TNF)-α and interleukin (IL)-6 expression levels, and increases the pro-inflammatory signaling pathways such as nuclear factor-κB (NF-κB) and mitogen-activated protein kinase (MAPK) [3,4].

Keratinocytes consist of 90% of the epidermis cells with five layers, which comprise the corneum (horny layer), lucidum (clear layer), granulosum (granular layer), spinosum (prickle cell layer), and basale (basal layer) [5]. Keratinocytes secrete pro-inflammatory cytokines, such as IL-1, IL-6, TNF-α, and interferon (IFN)-γ during the progression of their inflammatory process [6]. Specifically, TNF-α and IFN-γ release type-II T-helper cells (Th2)-mediated chemokines like thymus, activated-regulated chemokine (TARC/CCL17) and macrophage-derived chemokine (MDC/CCL22) that are members of the CC chemokine subfamily, and bind to CC chemokine receptor 4 (CCR4) [7,8]. TNF-α and IFN- γ stimulation of keratinocytes has been reported to induce the increase of pro-inflammatory cytokines and chemokines that work synergistically in the primary human keratinocytes cell line, HaCaT cells [7]. Th-2-associated chemokines have been recognized as a crucial mediator in chronic skin disease, suggesting that the regulation of TARC/CCL17 and MDC/CCL22 in keratinocytes may be used as an effective therapeutic target.

The development of inflammation causes skin damage and aging, including the creation of wrinkles due to reduced elasticity. The presence of wrinkles is closely related to decreased collagen production, and skin extracellular matrix (ECM), such as elastin, elastic fibers, and gelatin fibers [9,10]. The dermis ECM, which is produced by a fibroblast cell, is composed of collagen [11]. In particular, acute inflammation can lead to the existence of matrix metalloproteinase MMP-8, and the degradation of type I collagen [12]. Anti-wrinkle strategies have included decreased skin inflammation, increased collagen content, and inhibition of MMP-1 and -8 activity, which are known to degrade collagen [13].

Annexin A1 (AnxA1), a 37-kDa protein of the annexin family, is a glucocorticoid-regulated protein that has shown potent anti-inflammatory effects mediating acute, chronic, and systemic inflammation [14]. Several reported studies have suggested that AnxA1 is a crucial endogenous regulator, which respond to chemical insults, injury, and pro-inflammatory cytokines [15,16,17]. In the skin allograft model, AnxA1 mimic peptide (Ac2-26) decreases tissue injury and neutrophil infiltration through anti-inflammatory action [14]. Similarly, although various reported studies have demonstrated the anti-inflammatory effects of AnxA1 and its mimetic peptides, such as Ac2-26, only a few reports have studied the activity of AnxA1 in skin inflammatory processes well and there have been no studies on the administration of Ac2-26 in TNF-α/IFN-γ-stimulated human keratinocytes [18]. In addition, there has been no research reported on the wrinkle-related protein expression of Ac2-26 in human skin fibroblast cell lines.

In the current study, we examined the anti-inflammatory and collagen degradation effects of Ac2-26 in human keratinocyte and fibroblast cells, and further investigated its mechanisms.

## 2. Materials and Methods

### 2.1. Cell Culture and Reagents

The human keratinocyte cell line HaCaT and human fibroblast cell line Detroit 551 was cultured in Dulbecco’s modified Eagle’s medium (DMEM; Gibco, Thermo Fisher Scientific, Inc., Waltham, MA, USA) and Minimum Essential Medium (MEM; Gibco) containing 10% heat-inactivated fetal bovine serum (FBS; Gibco) and supplemented with 100 U/mL penicillin and 100 μg/mL streptomycin (Thermo Fisher Scientific, Inc., Waltham, MA, USA), respectively. The cells were incubated at 37 °C in a humidified atmosphere containing 5% CO_2_.

Ac2-26, annexin A1 protein mimetic peptide, were purchased from Tocris Bioscience (Missouri, UK). Recombinant human tumor necrosis factor (TNF)-α and interferon (IFN)-γ were obtained from Gibco and enzynomics (Daejeon, Republic of Korea), respectively.

### 2.2. Cell Viability Assay

Cell viability was analyzed using 3-(4,5-dimethylthiazol-2-yl)-2,5-diphenyltetrazolium bromide (MTT). Both cells were seeded at a density of 5 × 10^4^ cells per well in 48-well plates, and grown for 18 h. After treatment with the indicated concentrations of Ac2-26 with or without 10 ng/mL each of TNF-α and IFN-γ, cells were incubated for 24 h. The 0.5% MTT solution was added to each well, and the cells incubated for 2 h at 37 °C in incubator. The insoluble formazan was solubilized in DMSO, and then absorbance was measured at 540 nm by PowerWave HT microplate spectrophotometry (BioTek, Winooski, VT, USA).

### 2.3. Chemokine and Cytokine Analysis

HaCaT cells were incubated in a 48-well plate, and treated with Ac2-26 with or without TNF-α/IFN-γ for 24 h. To remove cell debris, the cell culture supernatant was centrifuged at 2000× *g* for 10 min. The MDC enzyme-linked immunosorbent assay (ELISA) kit was purchased from Abcam (Cambridge, UK), and IL-8 and IL-6 were purchased from Abbkine (Wuhan, China). The amounts of MDC and IL-8 in cell culture supernatant were analyzed using each ELISA kit, based on the manufacturer’s instructions. The absorbance was measured at 450 nm by PowerWave HT microplate spectrophotometry (BioTek, Winooski, VT, USA).

### 2.4. Western Blot Analysis

HaCaT cells were treated with the indicated concentration of Ac2-26 with TNF-α or IFN-γ for 24 h. Detroit 551 cells were treated with the indicated concentration of Ac2-26 for 24 h. Then the incubated cells were lysed using RIPA buffer (iNtRON Biotechnology, Gyeonggi, South Korea) containing a protease inhibitor cocktail and a phosphatase inhibitor (Thermo Fisher Scientific). The protein quantification of each cell lysate sample was measured using BCA assay (Thermo Fisher Scientific), according to the manufacturer’s instructions. Equal volumes of protein (~20 μg) were separated on (8–12)% SDS-polyacrylamide gel electrophoresis (SDS-PAGE), and then transferred to a polyvinylidene fluoride (PVDF) membrane (Immunobilon-P, 0.45 mm; Millipore, Billerica, MA, USA), using the semi-dry transfer system (Atto Corp., Tokyo, Japan). The membranes were blocked with 5% bovine serum albumin (BSA) in tris-buffered saline containing 1% Tween 20 (TBS-T, pH 7.4) at room temperature (RT) for 1 h, followed by incubation overnight at 4 °C with a 1:1000 dilution of the respective primary antibody. The membranes were washed five times with TBS-T for 10 min each at RT, and then incubated with a horseradish peroxidase (HRP)-conjugated secondary antibody for 2 h at RT. The membranes were then rewashed five times using TBS-T, detected by ECL reagent (Bio-Rad, Hercules, CA, USA), and analyzed using Image Lab 4.1 (Bio-rad) program. The densitometry analysis using Image J software (U.S. National Institutes of Health, Bethesda, MD, USA) of each of the protein bands was normalized according to the β-actin expression.

### 2.5. RNA Isolation and Quantitative Real-Time PCR

The Total RNA content was isolated using the Trizol^®^ reagent (Thermo Fisher Scientific, Inc., Waltham, MA, USA), and the concentration of RNA was measured by spectrophotometry (ND-1000; Thermo Fisher Scientific). Total RNA (1 μg) was reverse-transcribed into cDNA using the iScript™ cDNA synthesis kit (Bio-Rad Laboratories, Inc., Alfred Nobel Drive, Herculus, CA, USA), and qPCR was performed using AccuPower^®^ 2 × Greenstar™ qPCR Mastermix (Bioneer Corporation) and a CFX384 Real Time PCR Detection system (Bio-Rad Laboratories, Inc., Alfred Nobel Drive, Herculus, CA, USA), according to each manufacturer’s protocol. The primer sequences were as follows: IL-1β sense, 5′-CTGTCCTGCGTGTTGAAAGA-3′ and anti-sense, 5′-TTGGGTAATTTTTGGGATCTACA-3′; IL-6 sense, 5′-GCAGAAAACAACCTGAACCTT-3′ and anti-sense, 5′-ACCTCAAACTCCAAAAGACCA-3′; IL-8 sense, 5′- AGGGTTGTGGAGAAGTTT-3′ and anti-sense, 5′-GGCATCTTCACTGATTCTTG-3′; TNF-α sense, 5′-GACAAGCCTGTAGCCCATGTTGTA-3′ and anti-sense, 5′-CAGCCTTGCCCCTTGAAGA-3′.

MDC sense, 5′- CTACAGACTGCACTCCTGGTTGTC-3′ and anti-sense, 5′-GCCTGCCTCAGTTGCTTGA-3′; TARC sense, 5′-ATGGCCCCACTGAAGATGCT-3′ and anti-sense, 5′-TGAACACCAACGGTGGAGGT-3′; β-actin sense, 5′-TTCTACAATGAGCTGCGTGTGG-3′ and anti-sense, 5′-GTGTTGAAGGTCTCAAACATGAT-3′. The relative expressions of the mRNA were obtained and analyzed by Bio-Rad CFX Manager Version 3.1 software using the 2^−ΔΔCq^ method. The expression levels of each mRNA quantified were normalized against the expression of β-actin as reference gene.

### 2.6. Statistical Analysis

All experimental results are expressed as ± standard error of the mean (SEM) of at least triplicate samples using GraphPad Prism software (version 5.02; GraphPad Software, Inc., San Diego, CA, USA). Significant differences were calculated by one-way factorial analysis of variance (ANOVA) followed by Dunnett’s test. *p*-values < 0.05 were considered statistically significant.

## 3. Results

### 3.1. Effects of Ac2-26 on HaCaT Cells Cytotoxicity

**Figure 1:**
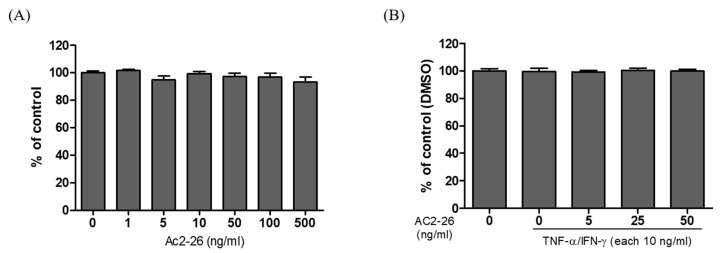
Cytotoxic effect of annexin A1 derived peptide, Ac2-26 on human keratinocyte, HaCaT cells. The cells were pretreated with or without TNFα/IFN-γ (each 10 ng/mL), and then subsequently treated with the indicated concentration of Ac2-26 at 37 °C for 24 h. 3-(4,5-dimethylthiazol-2-yl)-2,5-diphenyltetrazolium bromide (MTT) assay was carried out to measure the cytotoxic levels. (**A**) Effect of Ac2-26 on cell viability in HaCaT cells. (**B**) Effect of Ac2-26 on TNFα/IFN-γ-induced cell viability in HaCaT cells. Data are presented as the mean ± SEM of three independent experiments.

To identify the cytotoxicity, HaCaT cells were treated with the indicated concentration of Ac2-26 for 24 h. The results showed that Ac2-26 had no significant cytotoxicity on HaCaT cells, even after 24 h up to 500 ng/mL (Figure 1A). We also co-treated with Ac2-26 and 10 ng/mL of TNF-α or IFN-γ to investigate the non-toxic dose of Ac2-26. The co-treatment ranging from 5 to 50 ng/mL of Ac2-26 with TNF-α or IFN-γ was not cytotoxic on HaCaT cells (Figure 1B). Thus, we used Ac2-26 concentration of (5, 25, and 50) ng/mL for subsequent experiments.

### 3.2. Effects of Ac2-26 on the Production of TNF-α/IFN-γ-Stimulated Inflammatory Chemokines and Cytokines in HaCaT Cells

**Figure 2:**
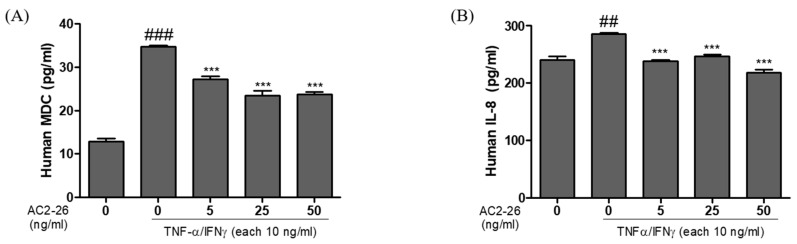
Inhibitory effect of Ac2-26 on TNFα/IFN-γ-induced chemokine production in HaCaT cells. HaCaT cells were pre-treated with TNFα/IFN-γ (each 10 ng/mL), and then subsequently treated with Ac2-26 of (0, 5, 25, and 50) ng/mL for 24 h. The levels of chemokine production were measured using ELISA. (**A**) Effect of Ac2-26 on TNFα/IFN-γ-induced macrophage-derived chemokine (MDC) production in HaCaT cells. (**B**) Effect of Ac2-26 on TNFα/IFN-γ-induced IL-8 production in HaCaT cells. Data are presented as the mean ± SEM of three independent experiments. ^###^ *p* < 0.001 vs. untreated group; ^##^ *p* < 0.01 vs. untreated group; *** *p* < 0.001 vs. TNF-α/IFN-γ only treated group.

To investigate the inhibitory effect of Ac2-26 in HaCaT cells on inflammation, we measured the expression of pro-inflammatory chemokines and cytokines. The TNF-α/IFN-γ treatment significantly increased the expression of MDC, TARC, IL-1β, and IL-6, compared with the control group, in HaCaT cells. However, the co-treatment group with Ac2-26 and TNF-α or IFN-γ significantly inhibited the production of these chemokines, compared with the only TNF-α- or IFN-γ-treated group (Figure 2A,B). The results show that Ac2-26 inhibited the production of chemokine MDC and IL-8 in a dose-dependent manner.

### 3.3. Inhibitory Effect of Ac2-26 on Chemokines mRNA Expression in TNF-α/IFN-γ-Stimulated HaCaT Cells

**Figure 3:**
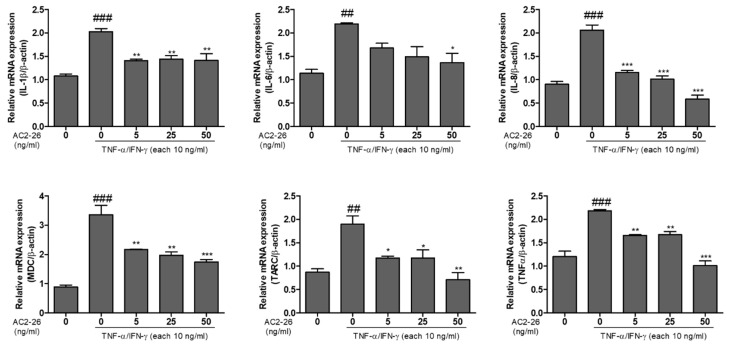
Inhibitory effect of Ac2-26 on the TNFα/IFN-γ-induced mRNA expression of cytokines and chemokines in HaCaT cells. HaCaT cells were pre-treated with TNFα/IFN-γ (each 10 ng/mL), and then subsequently treated with Ac2-26 of (0, 5, 25, and 50) ng/mL for 24 h. The mRNA expression levels of the indicated cytokines and chemokines (IL-1β, IL-6, IL-8, MDC, TARC, and TNF-α) were measured using RT-qPCR analysis. Each mRNA expression level was normalized against β-actin as the control. Data are presented as the mean ± SEM of three independent experiments. ^###^ *p* < 0.001 vs. untreated group; ^##^ *p* < 0.01 vs. untreated group; *** *p* < 0.001 vs. TNF-α/IFN-γ only treated group; ** *p* < 0.01 vs. TNF-α/IFN-γ only treated group; * *p* < 0.05 vs. TNF-α/IFN-γ only treated group.

We investigated the inhibitory effects of Ac2-26 on pro-inflammatory chemokine mRNA levels. The expression levels of IL-1β, IL-6, IL-8, MDC, TARC, and TNF-α genes were determined using quantitative real-time PCR. Then, as shown in Figure 3, stimulation with TNF-α/IFN-γ increased IL-1β, IL-6, IL-8, MDC, TARC, and TNF-α mRNA levels in HaCaT cells. As a result, these were significantly inhibited by Ac2-26 treatment, suggesting that Ac2-26 treatment can suppress the inflammatory response in TNF-α/IFN-γ stimulated HaCaT cells.

### 3.4. Effects of Ac2-26 on MAPK Phosphorylation in TNF-α/IFN-γ-Treated HaCaT Cells

**Figure 4:**
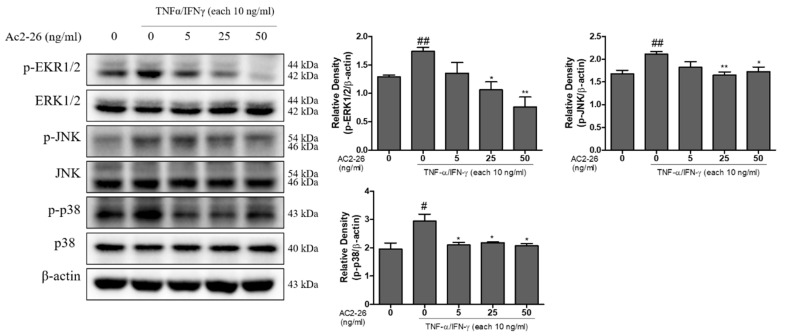
Inhibitory effect of Ac2-26 on the TNFα/IFN-γ-induced protein expression of MAPK phosphorylation in HaCaT cells. HaCaT cells were pre-treated with TNFα/IFN-γ (each 10 ng/mL), and then subsequently treated with Ac2-26 of (0, 5, 25, and 50) ng/mL for 24 h. The expression levels of the indicated proteins (p-ERK1/2, ERK1/2, p-JNK, JNK, p-p38, and p38) were measured using Western blot analysis. The relative expression of p-ERK1/2, p-JNK, and p-p38 ratios were quantified by densitometry. β-actin was used as the loading control. Data are presented as the mean ± SEM of three independent experiments. ^##^ *p* < 0.01 vs. untreated group; ^#^ *p* < 0.05 vs. untreated group; ** *p* < 0.01 vs. TNF-α/IFN-γ only treated group; * *p* < 0.05 vs. TNF-α/IFN-γ only treated group.

MAPK pathways play a crucial role in the regulation of pro-inflammatory molecules on cellular responses [19]. To determine the relevance of MAPK pathway with Ac2-26 in HaCaT cells, we examined the effects of Ac2-26 on the phosphorylation of JNK, p38, and ERK proteins. The treatment of TNF-α/IFN-γ significantly induced the activation of MAPK. Treatment with Ac2-26 significantly suppressed the phosphorylation of JNK, p38, and ERK, compared with the only TNF-α- or IFN-γ-treated group (Figure 4). The relative densities of the blots were quantified based on their densitometry. These findings suggest that Ac2-26 induces anti-inflammatory effects by regulating the activation of MAPK pathway.

### 3.5. Effects of Ac2-26 on NF-κB Signaling and JAK 2/STAT 3 Phosphorylation in TNF-α/IFN-γ-Treated HaCaT Cells

**Figure 5:**
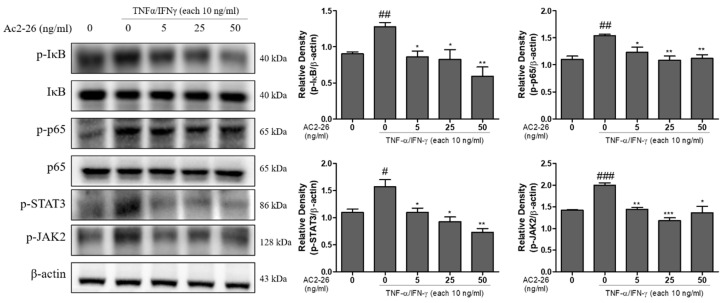
Inhibitory effect of Ac2-26 on the TNFα or IFN-γ-induced protein expression of NF-kB signaling and STAT3 or JAK2 phosphorylation in HaCaT cells. HaCaT cells were pre-treated with 10 ng/mL each of TNFα or IFN-γ, and then subsequently treated with Ac2-26 of (0, 5, 25, and 50) ng/mL for 24 h. The expression levels of the indicated proteins (p-IκB, IκB, p-p65, p65, p-STAT3, and p-JAK2) were measured using Western blot analysis. The relative expression of p-IκB, p-p65, p-STAT3, and p-JAK2 were quantified by densitometry. β-actin was used as the loading control. Data are presented as the mean ± SEM of three independent experiments. ^###^ *p* < 0.001 vs. untreated group; ^##^ *p* < 0.01 vs. untreated group; ^#^ *p* < 0.05 vs. untreated group; *** *p* < 0.001 vs. TNF-α/IFN-γ only treated group; ** *p* < 0.01 vs. TNF-α/IFN-γ only treated group; * *p* < 0.05 vs. TNF-α/IFN-γ only treated group.

The NF-κB and JAK-STATs signaling pathways play pivotal roles in inflammatory responses [20]. We investigated the inhibitory effect of Ac2-26 on NF-κB and JAK2/STAT3 signaling pathways by Western blot. These results showed that TNF-α/IFN-γ markedly induced the phosphorylation of IκB-α, p65, STAT3, and JAK2 in HaCaT cells, and this effect was inhibited by Ac2-26 treatment (Figure 5). The relative densities of the blots were quantified, based on their densitometry. These results strongly suggest that Ac2-26 markedly prevented NF-κB and JAK2/STAT3 activity.

### 3.6. Expression of Collagen on Ac2-26 Treated Human Fibroblast Cells

**Figure 6:**
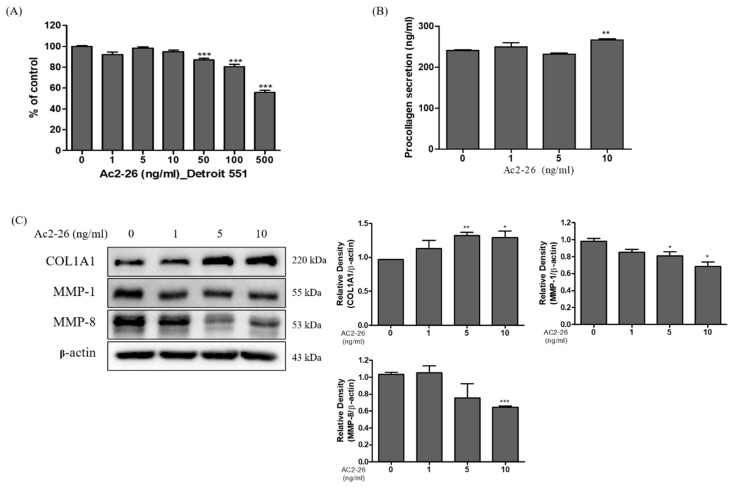
Expression of collagen on Ac2-26 treated human fibroblast Detroit 551 cells. The cells were treated with the indicated concentration of Ac2-26 at 37 °C for 24 h. MTT assay was carried out to measure the cytotoxic levels. (**A**) Effect of Ac2-26 on the cell viability of Detroit 551 cells. (**B**) Effect of Ac2-26 on the production of procollagen secretion in Detroit 551 cells measured by ELISA. (**C**) Western blot analysis on the expression levels of protein markers COLIA1, MMP-1, and MMP-8 on Ac2-26 treated Detroit 551 cells. *** *p* < 0.001 vs. untreated group; ** *p* < 0.01 vs. untreated group; * *p* < 0.05 vs. untreated group.

The anti-inflammatory effects of compounds such as hesperidin have been reported to prevent skin thickening, and wrinkle formation [21]. Therefore, we investigated the anti-wrinkle related proteins on Ac2-26 treated human fibroblast cells, Detroit 551. The cell viability assay results (Figure 6A) showed no cytotoxicity at (1, 5, and 10) ng/mL concentrations. Therefore, we used these concentrations in further experiments. Wrinkle formation is closely related to the reduction of collagens in dermal skin [22]. Based on these previous studies, the induction of collagen can lead to effective treatment for anti-wrinkle conditions. We checked procollagen secretion in Ac2-26 treated Detroit 551 cell medium. These results showed that Ac2-26 treatment increased the level of procollagen, suggesting that Ac2-26 treatment can induce collagen synthesis (Figure 6B). In addition, we identified the expression levels of protein collagenase, including MMP-1, MMP-8, and COL1A1. Figure 6C shows the results, which clearly reveal that Ac2-26 significantly inhibited the protein levels of MMP-1 and MMP-8, and increased the protein level of COL1A1. The relative densities of the blots were quantified based on their densitometry. These data suggest that Ac2-26 can decrease collagen degradation and induce collagen synthesis in Detroit 551 cells.

## 4. Discussion

Skin aging prevention has gained significant interest, both clinically and cosmetically. In the present study, we examined the anti-inflammatory and anti-wrinkle effect of Ac2-26, AnxA1 mimetic peptide, which is known as an anti-inflammatory protein in skin keratinocyte and fibroblast cells.

One of the main characteristic features of skin inflammation is the infiltration into the inflamed skin region of various immune cells, including monocytes [23]. The dysregulation of the cytokines/chemokines and other adhesion molecules improves the immune cell infiltration into the site of skin inflammation [24]. Keratinocytes, when stimulated with inflammatory cytokines, such as tumor necrosis factor-alpha (TNF-α) and interferon-γ, can express adhesion molecules, such as various cytokines/chemokines [25]. Dysregulated inflammatory responses, such as inflammatory diseases, contribute to multiple pathological disorders. In the area of inflamed skin, up-regulated pro-inflammatory mediators play the role of a vital mechanism. Hence, the down-regulated pro-inflammatory skin mediators present an important strategy for modulating various inflammatory skin diseases [26,27]. Keratinocytes, by producing pro-inflammatory chemokines, play an important role in inflammatory skin disease. Many studies have reported that TNF-α/IFN-γ, Th-2 related chemokines, treatment increases the production of chemokines in HaCaT cells. In the present study, the TNF-α/IFN-γ treatment group upregulated the release of MDC and IL-6, and mRNA expression levels of cytokines and chemokines, such as IL-1β, IL-6, Il-8, MDC, TARC, and TNF-α (Figure 2; Figure 3).

Consistent with the current findings, previous studies revealed that TNF-α/IFN-γ-stimulation activates multiple intracellular signaling pathways, including mitogen-activated protein kinases (MAPKs), NF-κB and STAT-1/JAK-2 pathways [28,29]. MAPKs and STAT/JAK signaling pathways have been shown to participate in controlling the development of chemokines in HaCaT cells. These cascades play an important role in immune response, and control the signaling pathway to inflammation [30]. In the present study, Ac2-26 suppresses chemokines secretion and mRNA expression levels in TNF-α/IFN-γ-stimulated HaCaT cells. In addition, Ac2-26 appears to control chemokine results by inhibition of the activation of MAPK, NF-κB, as well as STAT/JAK signaling pathway. Several naturally derived products or synthetics with anti-inflammatory effects can be used to treat inflammatory skin diseases, such as atopic dermatitis, urticaria, and eczema. Atopic dermatitis is one of the chronic inflammatory skin diseases worldwide [29]. In the area of skin lesions in chronic atopic dermatitis, immune cells secrete the chemokines TNF-α and IFN-γ [31]. Based on the current results, we suggest that Ac2-26 can not only alleviate the response of atopic dermatitis by regulating chemokines, but also suppress the activation of MAPK, NF-κB, as well as STAT/JAK signaling pathways in TNF-α/IFN-γ stimulated HaCaT cells.

Inflammation in skin fibroblast cells can be induced by environmental influences, such as UV exposure and environmental hazard [32]. These factors cause accumulative changes in skin fibroblast microenvironments and lead to skin aging [9,33]. In particular, a close correlation was found with the up-regulation of inflammatory cytokines, such as IFN-γ, TNF-α, IL-6, and TGF-β, and damaged skin fibroblast elastic fibers, suggesting that a loss of skin elasticity subsequently leads to wrinkle formation [34]. Therefore, skin inflammation can play a pivotal role in skin remodeling. Collagen occurs at the highest concentration in the skin’s dermal layer, accounting for (70–80)% of the total dry weight, and plays a role in maintaining the skin [35]. The proportion of type I collagen in dermis decreases with aging of the skin internally and externally [10]. In the current study, we showed the increase of pro-collagen secretion and collagen generation induced by Ac2-26 treatment. Among the major enzymes in skin wrinkles, MMPs destroy structural dermis proteins, including collagen and elastin, to increase wrinkles, and facilitate aging [36]. We identified that the MMP-1 and -8 protein expression were dose-dependently downregulated in Ac2-26 treated Detroit 551 cells. Thus, collectively, the current study demonstrates a preliminary approach that Ac2-26 possesses anti-inflammatory and anti-wrinkle effect and, with further validations, could have potential in the treatment of skin-related diseases.

## 5. Conclusions

**Figure 7:**
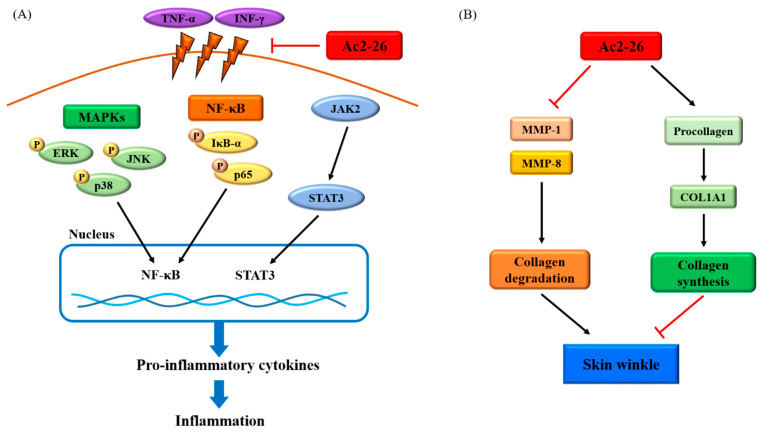
Schematic of the pathway of Ac2-26 effect: (**A**) the mechanism of the anti-inflammatory responses of Ac2-26 in HaCaT cells. This pathway represents the phosphorylation of IκB-α, p65, STAT3, and JAK2 induced by TNF-α/IFN-γ, and was inhibited upon Ac2-26 treatment in HaCaT cells. (**B**) Anti-wrinkle effect upon the inhibition of Ac2-26 in Detroit cells. This pathway represents the inhibition of the protein levels - MMP-1 and MMP-8 leading to collagen degradation, and increases the protein level of COL1A1 leading to collagen synthesis. (Tumor necrosis factor-α; TNF-α, interferon-γ; IFN-γ, mitogen-activated protein kinase; MAPK, nuclear factor- κB; NF-κB, matrix metalloproteinase-1; MMP-1, matrix metalloproteinase-8; MMP-8, collagen alpha-1; COL1A1).

In conclusion, the present data show that Ac2-26 inhibited the expression and secretion of TNF-α/IFN-γ-induced pro-inflammatory chemokines through a blockade of the MAPK, NF-κB, and STAT/JAK pathways. In addition, treatment of human fibroblast skin cells with Ac2-26 upregulated pro-collagen secretion and collagen generation, and down-regulated MMP proteins. Taken together, Ac2-26 may be applicable as a cosmeceutical agent for the regulation of inflammation and wrinkle formation in the skin, as summarized in Figure 7.
